# Generation of fast photoelectrons in strong-field emission from metal nanoparticles

**DOI:** 10.1515/nanoph-2024-0719

**Published:** 2025-04-04

**Authors:** Erfan Saydanzad, Jeffrey Powell, Tim Renner, Adam Summers, Daniel Rolles, Carlos Trallero-Herrero, Matthias F. Kling, Artem Rudenko, Uwe Thumm

**Affiliations:** J. R. Macdonald Laboratory, Department of Physics, 5308 Kansas State University, Manhattan, 66506, Kansas, USA; INRS, Énergie, Matériaux et Télécommunication, Varennes, J3X 1P7, Québec, Canada; Department of Physics, University of Connecticut, Storrs, 06269, CT, USA; Department of Physics, Goethe University Frankfurt, Frankfurt am Main, 60323, Germany; SLAC, National Accelerator Laboratory, Menlo Park, 94025, CA, USA; Department of Applied Physics, Stanford University, Stanford, 94305, CA, USA

**Keywords:** plasmonics, nanoparticle, plasmonic-field enhancement, strong-field ionization, photoelectron imaging, electron source

## Abstract

We investigated the generation and control of fast photoelectrons (PEs) by exposing plasmonic nanoparticles (NPs) to short infrared (IR) laser pulses with peak intensities between 10^12^ and 3 × 10^13^ W/cm^2^. Our measured and numerically simulated PE momentum distributions demonstrate the extent to which PE yields and cutoff energies are controlled by the NP size, material, and laser peak intensity. For strong-field photoemission from spherical silver, gold, and platinum NPs with diameters between 10 and 100 nm our results confirm and surpass extremely high PEs cutoff energies, up to several hundred times the incident laser-pulse ponderomotive energy, found recently for gold nanospheres [Saydanzad et al., Nanophotonics **12**, 1931 (2023)]. As reported previously for dielectric NPs [Rupp et al., J. Mod. Opt. **64**, 995 (2017)], at higher intensities the cutoff energies we deduce from measured and simulated PE spectra tend to converge to a metal-independent limit. We expect these characteristics of light-induced electron emission from prototypical plasmonic metallic nanospheres to promote the understanding of the electronic dynamics in more complex plasmonic nanostructures and the design of nanoscale light-controlled plasmonic electron sources for photoelectronic devices of applied interest.

## 1 Introduction

Photoemission from metallic nanostructures is of fundamental and practical relevance for attosecond field emission [1], [2], attosecond streaking spectroscopy of metal NPs [3], [4], efficient harmonic up-conversion [5], [6], femtosecond time-resolved scanning tunneling microscopy and spectroscopy [7], [8], [9], electron-impact spectroscopy [10], [11], and the development of compact electron sources [12]. Here, we demonstrate that prototypical plasmonic nanospheres, when subjected to intense IR-laser pulses, emit PEs across a broad kinetic energy spectrum, driven by a complex dynamical interplay of electronic and photonic interactions. Over the past two decades, the pronounced optical properties and plasmonic response of conduction electrons in metal NPs in the IR to visible frequency range have been extensively studied [13], [14]. The excitation of localized surface-charge plasmons (LSP) at nano-structured surfaces influences the particles’ light absorption, reflection, and skin depths [15] and creates a nanoplasmonic field near the NP surface that can significantly amplify the incident laser electric field [16], [17], [18]. Nanoplasmonic field enhancement was predicted theoretically to importantly increase the cutoff energy in the photoionization of xenon atoms near metal NPs [19]. The LSP resonance frequency of metal NPs can be tuned from IR to visible frequencies by altering the NP shape, size, composition, and dielectric environment [13], [14], [20], [21], [22]. This tunable enhancement of light absorption and scattering is crucial for advanced diagnostic methods, such as surface-enhanced Raman spectroscopy [23], [24], time-resolved nanoplasmonic-field microscopy [17], [25], [26], [27], polariton chemistry [28], and biomedical and chemical sensing [29], [30], [31], [32], [33].

In the present work, we use velocity-map-imaging (VMI) spectroscopy to investigate strong-field electron emission from metal NPs. By measuring and numerically modeling VMI spectra resulting from intense IR-laser pulses, we validate for metal NPs a recent extension [26] of the three-step model for atomic strong-field ionization [34]. VMI spectroscopy projects PE momentum distributions onto a 2-dimensional detector plane and is widely utilized to study intense-light interactions with atoms and molecules [35], [36], [37]. Over the past decade, this method has been used to explore strong-field photoemission from isolated NPs with intense linearly polarized laser pulses [38], [39], [40], [41]. Upon emission from atoms and molecules, PEs can gain significant energy while propagating in the oscillating strong electric field of a laser pulse [42]. It is well known that for ‘direct’ emission (where the PE is not driven back to the residual ion by the external light field) from gaseous atomic targets by linearly polarized laser pulses, PEs gain up to 2 *U*_*p*_(*I*_0_) in kinetic energy, while ‘rescattered’ PEs (that are driven back by the laser electric field to elastically scatter off the residual ion) can accumulate up to 10*U*_*p*_(*I*_0_) [43], [44], [45], [46]. The ponderomotive energy *U*_*p*_(*I*_0_) = *I*_0_/(4*ω* ^2^) is the cycle-averaged quiver energy of a free electron in the incident laser field of frequency *ω* and peak intensity *I*_0_. Unless otherwise stated, atomic units are used throughout this work.

For strong-field PE emission from metallic nanotips and isolated clusters, PE cutoff energies for direct and rescattered photoemission were found previously to exceed 30 *U*_*p*_(*I*_0_) [47] and 15 *U*_*p*_(*I*_0_) [48], respectively. Higher cutoff energies of about 50 *U*_*p*_(*I*_0_) [40] and 140 *U*_*p*_(*I*_0_) [22] were measured for dielectric SiO_2_ and Fe_3_O_4_ NPs, respectively. Strong-field photoemission from isolated dielectric SiO_2_ NPs by intense 25 fs 780 nm linearly polarized laser pulses was recently measured for different NP sizes and laser intensities [39]. Compared to atomic targets, strong-field emission from *metallic* plasmonic NPs was recently found experimentally [15], [49] and theoretically [26], [49] to yield the most significant enhanced PE cutoff energies of several hundred *U*_*p*_(*I*_0_), due to substantial nanoplasmonic field enhancement of the incident ionizing laser pulse and a large number of emitted electrons. Caused by the strong plasmonic-near-field enhancement of the incident-laser electric field and PE correlation, the calculated cutoff energies for metal NPs surpass those typical from gaseous atoms and dielectric NPs by two and up to one order of magnitude, respectively [26], [49].

## 2 Methods

### 2.1 Experimental setup

**Figure 1:**
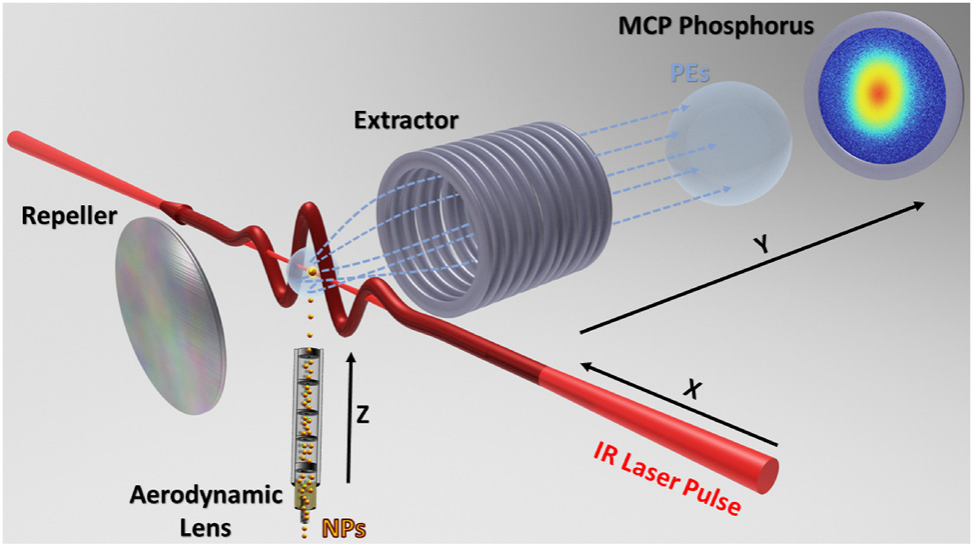
(Color online) Schematic of the NP source and velocity-map-imaging spectrometer setup. A beam of single, isolated NPs is injected into vacuum by an aerodynamic lens and intersects an intense linearly polarized 800 nm, 26 fs laser beam operated at a repetition-rate of 10 kHz. Emitted electrons are focused onto a microchannel plate (MCP) and phosphor assembly, from which a camera records the spatial distribution of PE hits for individual laser shots.

The experiments were carried out at the James R. Macdonald Laboratory at Kansas State University. The laser system, NP source, and VMI electron detection assembly is described in more detail in Refs. [39], [50]. Briefly, we used a Ti:Sapphire-based chirped pulse amplification (CPA) system generating 25 fs (10 optical cycles) full-width-half-intensity maximum (FWHIM) pulses with a central angular frequency of *ω* = 2.354 PHz (corresponding to a central wavelength of *λ* = 800 nm). The NP source consisted of an atomizer, dryer, and aerodynamic lens and injects single, isolated particles into the vacuum, where the beam of NPs intersects the focused intense laser beam [41], [50], [51], [52], [53]. As depicted in the sketch of the experimental setup Figure 1, PEs generated by strong-field ionization of NPs in the laser pulse, are projected onto the detector by the static electric field between the repeller and extractor of the VMI system, allowing for the recording of the 2D projection of the PE velocity distribution. The thick-lens, high-energy VMI spectrometer is capable of detecting PEs with kinetic energies up to 350 eV [54]. The colloid NP samples with low polydispersity and high purity were purchased from Cytodiagnostics Inc. (gold and silver NPs) and Nanocomposix (platinum NPs) [55], [56].

### 2.2 Laser-intensity characterization

For the laser intensity and PE momentum calibration, we employed the VMI spectrometer described above to measure above-threshold-ionization (ATI) PE momentum distributions from gaseous Xe atoms [57]. This measurement was done under the same experimental conditions as for the photoemission-from-NP experiments reported here. From the 2D projection of the PE momentum distributions, we determined the ponderomotive shift of the ATI combs. Based on the proportionality of the ponderomotive energy and peak intensity of the incident laser pulse, *I*_0_, we determined the intensities used in this work in the interval 10^12^ W/cm^2^ < *I*_0_ < 3 × 10^13^ W/cm^2^. We estimated the accuracy of the intensity calibration to be better than 15 % [50], [58].

### 2.3 Theoretical model

The high laser intensities we consider generate multiply ionized NPs [39], [51]. We numerically model PE emission from metallic NPs by IR-laser pulses with a Gaussian temporal profile. Propagating along the *x* axis and linearly polarized along the *z* axis, their electric field is given by

(1) $$\begin{array}{cc} {\vec{E}}_{\mathrm{inc}}\left(\vec{r},t\right) & =\sqrt{I_{0}}\exp\left[-2\ln2\frac{{\left(t-x/c\right)}^{2}}{\tau^{2}}\right]\\ & \;\times \exp\left[-i\omega \left(t-x/c\right)+i\psi \right]\;{\hat{e}}_{z}, \end{array}$$

where *τ* is the pulse length at FWHIM, *ω* the pulses’ central frequency, *ψ* the carrier-envelope phase, and *c* the speed of light in vacuum (Figure 1). During the laser - NP interaction, LSPs are excited and induce an inhomogeneous plasmonic field near the NP surface. Most significantly at the LSP resonance frequency [59], [60], electrons are excited to electronic states above the Fermi level. Sufficiently high laser intensities generate multiply ionized NPs [39], [51]. The incident laser pulse thus transiently polarizes the NP. Within the electric-dipole approximation, the corresponding transient induced plasmonic dipole moment, ${\vec{P}}_{pl}\left(t\right)=\varepsilon_{0}\alpha_{\mathrm{Mie}}\left(\omega \right){\vec{E}}_{\mathrm{inc}}\left(\vec{r},t\right)$ , gives raise to the plasmonic electric field [61]

(2) $$\begin{array}{cc} {\vec{E}}_{pl}\left(\vec{r},t\right) & =\frac{e^{\mathrm{ikr}}}{r}\left\{k^{2}\left[{\hat{e}}_{r}\times {\vec{P}}_{pl}\left(t\right)\right]\times {\hat{e}}_{r}\right.\\ & \;\left.+\left[3{\hat{e}}_{r}\left[{\hat{e}}_{r}\cdot {\vec{P}}_{pl}\left(t\right)\right]-{\vec{P}}_{pl}\left(t\right)\right]\left(\frac{1}{r^{2}}-\frac{ik}{r}\right)\right\}, \end{array}$$

where *k* = 2*π*/*λ* = *ω*/*c*. We calculate the complex NP polarizability, *α*_*Mie*_(*ω*), within Mie theory [62], [63], which restricts the applicability of Eq. (2) for nanospheres of radius *a* to size parameters *ka*⪅0.6 [64].

We describe strong-field ionization from metal NPs by extending the semi-classical three-step model (also known as the ‘simple-man model’) for atomic strong-field ionization to metal NPs [26]. Our extended three-step model consists of (1) electron release based on quantum-mechanical tunneling, (2) PE propagation from the NP surface to the detector by sampling over classical trajectories, and (3) PE rescattering and recombination at the NP surface. In comparison with gaseous atomic targets, each of these steps is significantly more intricate for metal NPs. This is due to the NPs’ more complex electronic structure, the added morphological structure, and the emission of a much larger number of electrons compounding the effects of PE - PE correlation, residual charges, and PE - nanoplasmonic-field interactions.

We represent the NPs’ static electronic structure in terms of the surface potential step *V*_0_ = *ɛ*_*F*_ + *φ*, given by the work function *φ* and Fermi energy *ɛ*_*F*_ for bulk metal [65]. During successive small time intervals, our dynamical numerical simulation divides the NP surface into small surface elements, which are modeled as square-well potentials. Bound PEs close to the NP surface are assumed to tunnel out in radial direction, driven by the radial component of the total electric field at the NP surface, $\vec{F}\cdot {\hat{e}}_{r}$ , where $\vec{F}={\vec{E}}_{\mathrm{inc}}+{\vec{E}}_{pl}+{\vec{F}}_{\mathrm{res}}$ . The residual-charge field ${\vec{F}}_{\mathrm{res}}$ results from the accumulation of positive residual charge on the NP during electron emission in preceding time intervals. We account for strong-field electron release from the NP by employing modified [26] Fowler-Nordheim tunneling rates [66]. Subsequently, we Monte Carlo sample over the initial phase-space distribution of released electrons and solve Newton’s equations of motion for the PE propagation outside the NP in the presence of all electric fields, $\vec{F}+{\vec{F}}_{e-e}$ , where ${\vec{F}}_{e-e}$ is the repulsive Coulomb electric field between PEs. In each laser half-cycle the direction of the incident-laser electric field changes, such that emitted PEs can be accelerated back toward the NP and either rescatter from or recombine at the NP surface. We include and numerically evaluate the effects of PE repulsion, residual positive charges on the NP, PE recollisions and recombinations at the NP surface, and nanoplasmonic enhancement of the incident-laser-pulse electric field. More details about the numerical model are given in Ref. [26] and the Supplementary Information of Ref. [49].

## 3 Experimental and simulation results

### 3.1 Photoelectron-momentum images

**Figure 2:**
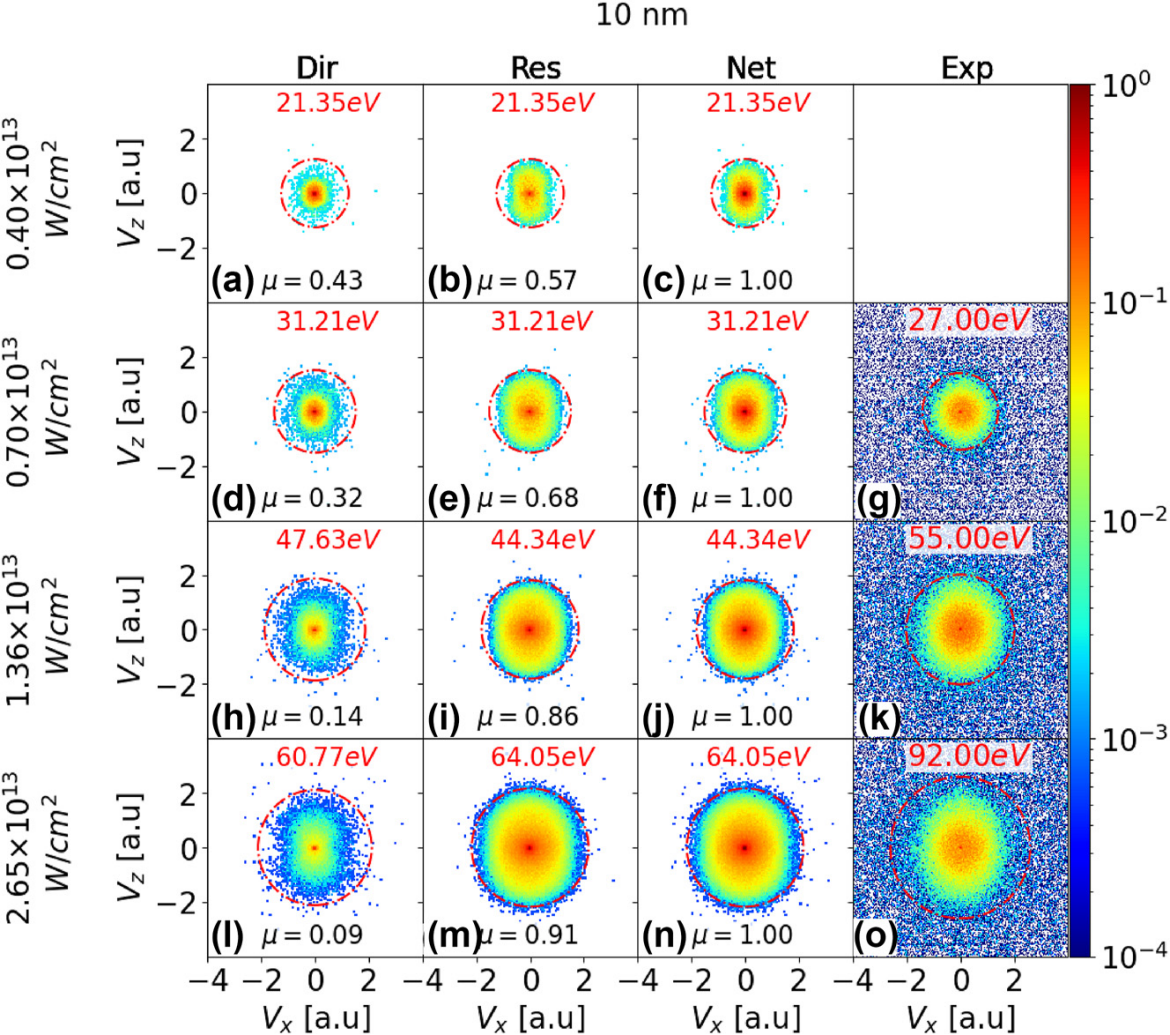
VMI PE spectra for strong-field ionization of silver nanospheres with 10 nm diameter and laser peak intensities of 4.0 × 10^12^, 7.0 × 10^12^, 1.36 × 10^13^, and 2.65 × 10^13^ W/cm^2^ (first - fourth row, respectively). Comparison of simulated direct (first column), rescattered (second column), and net (direct plus rescattered yields, third column) spectra with experimental spectra (fourth column). The laser-pulse length and wavelength are 25 fs FWHIM and 800 nm, respectively. In each row, calculated integrated PE yields for direct and rescattered emission, *μ*, are normalized to the integrated net yields in the third column. The red circle in each VMI map represents the photoemission cutoff. The cutoff energies are given in red above the VMI maps. No experimental data is available for 4.0 × 10^12^ W/cm^2^ peak intensity.

**Figure 3:**
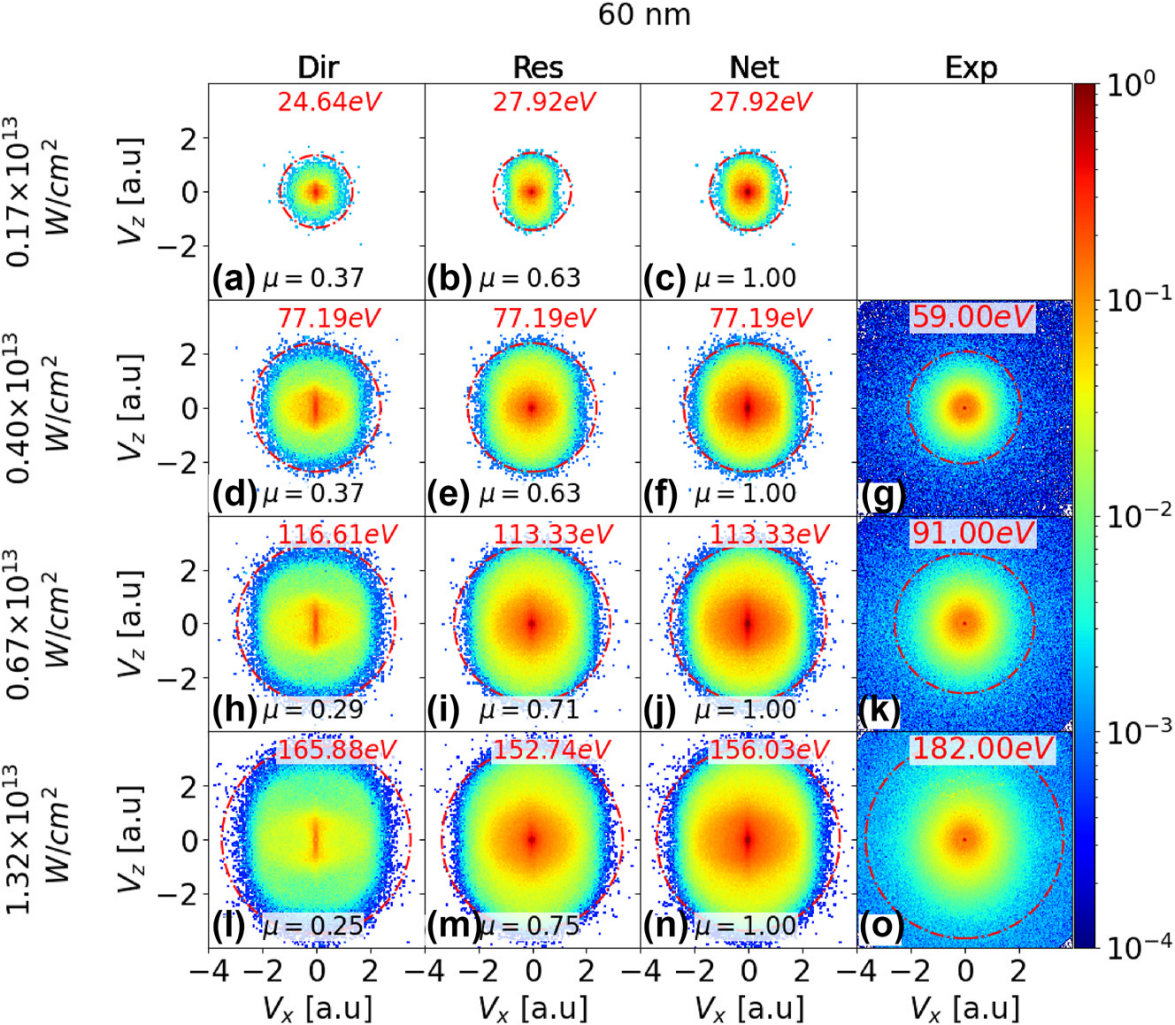
As Figure 2 for 60 nm diameter silver nanospheres and laser peak intensities of 1.7 × 10^12^, 4.0 × 10^12^, 6.7 × 10^12^, and 1.32 × 10^13^ W/cm^2^ (first – fourth row, respectively).

We start the presentation of our results with a comparison of laser-intensity and size-dependent measured and numerically simulated PE spectra for silver, gold, and platinum nanospheres. Figures 2, 3, and S1 display simulated and experimental VMI spectra for silver nanospheres with diameters of 10, 60, and 100 nm, respectively. The first, second, and third columns present simulated VMI spectra for the direct, rescattered, and net PE yields. The rows represent results for different peak laser intensities *I*_0_. Our numerical results in the third column are compared with our measured VMI spectra, shown in the fourth column for the three highest intensities. To facilitate a quantitative comparison between the direct and rescattered PE yields, we normalized the yields in each row to the corresponding net PE yield in the third column and display normalized integrated yields *μ* in all graphs.

For comparison with the simulated VMI spectra for silver NPs in Figures 2, 3, and S1, we provide in Figures S2, S3, and S4 of the Supplementary Information (SI) corresponding calculated spectra for gold NPs of identical diameter for the same peak intensities. Additional measured and simulated VMI spectra for gold NPs with different diameters and for other laser intensities than in the present work can be found in Ref. [49]. Figures S5 and S6 in the SI show a comparison of simulated and experimental VMI spectra for gold and platinum NPs with a diameter of 70 nm, respectively. For the examined transition metals, all simulated VMI spectra exhibit a slight elongation along the laser polarization direction, with PE cutoff energies and yields increasing with NP size and laser intensity. The high degree of isotropy is a direct result of the Coulomb repulsion between PEs and diffuse PE rescattering from the NP surface, as discussed for gold NPs in [26], [49].

We expect the yield parameter *μ* for rescattered PEs to consistently increase with both NP size and laser intensity: Higher laser intensities result in enhanced PE emission from the NP surface and higher residual charge accumulation on the NP. The accumulated residual charge enhances the yield of rescattered PEs. An increase in NP size provides a larger surface area for the laser - NP interaction, leading to increased PE emission and, consequently, enhanced PE rescattering. This effect is somewhat mitigated at larger sizes due to the, on average, larger distance between emitted PEs and accumulated residual charges.

The increase in yield with both NP size and intensity is nonlinear. An increasing residual charge on the NP tends to impede electron emission and, thus, to slow the growth of the electron yield with increasing laser intensity. This effect is comparable to the increase of the residual charge and work function of fullerene clusters during sequential electron capture by slow highly charged ionic projectiles [67], [68], [69]. Below, we shall refer to this observation as ‘*yield-saturation effect*’. It occurs with increasing laser peak intensity *I*_0_ at larger PE yields. We hold the underlying electronic dynamics accountable for the complex change of the branching ratio between direct and rescattered PE yields as a function of the laser intensity and NP size (cf., Figures 2, 3, S1, and SI).

At low intensities, the yield of rescattered PE increases relative to the direct-emission yield with the NP size, as is seen by comparing, e.g., Figures 2b and 3b. This yield increase is compatible with the larger effective surface area (geometrical cross section) for laser – NP interactions and electron rescattering. Consequently, an increase in NP size leads to higher PE yields and promotes rescattering. In contrast, the rescattering yield decreases for increasing NP size relative to direct emission at high intensities, as is illustrated by comparing, e.g., Figure 2(i) and 3(i) or Figure 2(m) and 3(m) for silver NPs. This indicates that now, at high intensity, the increase of the geometrical rescattering cross is less competitive in promoting rescattering over direct emission. The relative smaller chance for rescattering is compatible with the lower rate of residual-charge accumulation that is also deemed responsible for the yield-saturation (cf. Figure 5).

### 3.2 Cutoff energies and net PE yields

In this subsection we discuss laser-intensity and NP-size dependent PE cutoff energies and total yields for silver, gold, and platinum nanospheres. Extended exposure to the laser electric field leads to rescattering as the dominant mechanism for reaching the highest observable PE kinetic energies. The PE cutoff energy $E_{\text{cutoff}}^{R}$ is thus determined by rescattered PEs (denoted by the superscript ‘ *R*’). The effects of PE Coulomb repulsion, PE attraction by the built-up residual positive charge of the NP, and plasmonic field enhancement on $E_{\text{cutoff}}^{R}$ can be represented by the heuristic formula [49].

(3) $$E_{\text{cutoff}}^{R}=10\;U_{p}\left(I_{0}\right){\left[\eta_{\text{eff}}^{R}\left(a,I_{0}\right)+\omega t_{f}\eta_{C}^{R}\left(a,I_{0}\right)\right]}^{2}.$$

This expression describes the influence on photoemission of the plasmonic field enhancement near the NP in terms of the averaged enhancement factor $\eta_{\text{eff}}^{R}\left(a,I_{0}\right)<\eta \left(a,I_{0}\right)$ [64]. The effective enhancement factor $\eta_{\text{eff}}^{R}$ is expected to be smaller than the plasmonic field enhancement *η*(*a*, *I*_0_) due to the decreasing strength of the nanoplasmonic field for increasing polar angles and PE-distances and due to averaging over the PE dynamics. Repulsive PE – PE and attractive PE – residual-charge Coulomb interactions are represented within a simplified central-field picture, respectively, as the contributions $\eta_{e-e}^{R}\left(a,I_{0}\right)>0$ and $\eta_{\mathrm{res}}^{R}\left(a,I_{0}\right)>0$ to the effective Coulomb interaction factor

(4) $$\eta_{C}^{R}\left(a,I_{0}\right)=\eta_{e-e}^{R}\left(a,I_{0}\right)-\eta_{\mathrm{res}}^{R}\left(a,I_{0}\right).$$

**Figure 4:**
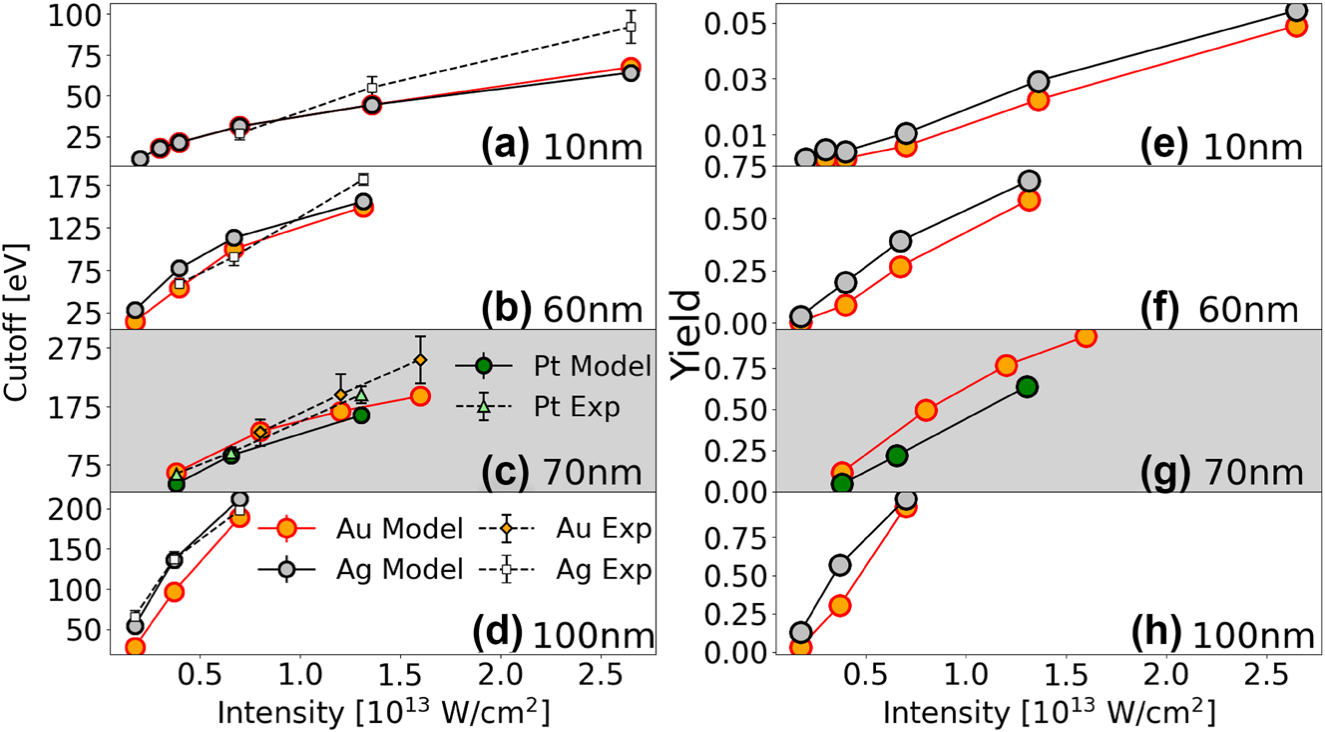
Simulated and experimental PE cutoff energies (a–d) and simulated yields (e–h) for gold, silver, and platinum nanospheres. (a,b,d,e,f,h) show PE cutoff energies and yields for gold and silver nanospheres with diameters of 10, 60, and 100 nm. (c,g) display PE cutoff energies and yields for gold and platinum nanospheres with a diameter of 70 nm. The gold – platinum comparisons is plotted against a gray background, for better distinction from the silver – gold results.

Our measured large cut-off energies indicate PE – PE interactions to dominate over PE –residual charge interactions for all intensities and sizes, such that $\eta_{C}^{R}\left(a,I_{0}\right)>0$ . For the laser parameters used in this study, the effective interaction time is determined at numerical convergence as *ωt*_*f*_ = 120.75 ≈ 2*ωτ*. Equation (3) and Table 1 explain qualitatively our experimental and simulated results in Figure 4 in terms of material characteristics and the NP size. The PE yield and cutoff energy tend to increase with decreasing work function, increasing plasmonic field enhancement, and increasing NP diameter [26], [49]. The leading factor *U*_*p*_(*I*_0_) in Eq. (3) is linear in the laser peak intensity *I*_0_. We further note that the magnitude of the linear shift of the peak position in ATI spectra measured from sharp metal tips [70] was previously attributed to the plasmonic field enhancement of the incident IR pulse [71], in support of the evidence we find for strong plasmonic effects on photoemission from metal NPs.

**Table 1:** Work function *φ* and calculated plasmonic field enhancement *η* for gold, silver, and platinum nanospheres of diameter 2*a*. The listed work functions are bulk values.

Material	2*a* [nm]	*φ* [eV]	*η*
Gold	10, 60, 70, 100	5.10	3.27, 3.42, 3.47, 3.65
Silver	10, 60, 100	4.26	3.23, 3.37, 3.59
Platinum	70	5.65	3.26

Figures 4a, b, and d present measured and simulated PE cutoff energies for gold and silver nanospheres. Figure 4c shows PE cutoff energies for platinum nanospheres with a diameter of 70 nm. We numerically simulated cutoff energies as the energy up to which 99.5 % of the net PE yield has accumulated [26], [49]. The experimental cutoff energies were extracted from the VMI maps as described in more detail in Refs. [39], [50].

Although experimental and simulated values are comparable in magnitude and show the same general trends in their size and intensity dependence, their overall agreement in Figure 4 is not perfect. We attribute the remaining discrepancies in part to the following uncertainties: With regard to the numerical simulation, a serious uncertainty derives from our implementation of approximate modified Fowler–Nordheim tunneling rates. While our simulated spectra are background-free and not adjusted for the experimental spectral detection efficiency, both background yields and detection uncertainties affect the measured data. The measured PE yields are subject to focal-volume averaging, such that for a given laser peak intensity, NPs exposed to a large range of intensities contribute to the PE yield. We partially offset this focal-volume effect by generating histograms of the number of PEs emitted per laser shot versus the number of laser shots. We then selected for our data analysis laser shots that yield the largest numbers of detected PEs, assuming they have illuminated NPs with intensities close to the laser peak intensity *I*_0_. PE counts associated with these preselected shots were added to compose the VMI maps shown in this work. From these maps the cutoff energies were defined where the PE yield becomes indistinguishable from the background [39], [50]. Other effects contributing to the measurement error in PE yields and energies are related to the (i) predominant emission of slow electrons overexposing the center of our MCP detector for the larger NPs and higher peak intensities used in the present work, (ii) NPs deviating from the ideal spherical shape and nominal size, and (iii) NP surfaces being coated with residual chemicals. Due to detection inefficiencies, the recorded PE yield is smaller than the actual yield. This difference is more significant for larger NPs and higher laser peak intensities.

Simulated PE yields for the NP materials and sizes in Figures 4a–d are shown in Figures 4e–h, respectively. For the laser parameters and targets in this study, comparing different materials of equal size shows that plasmonic field enhancements vary slightly for the considered metals (Table 1). PE yields and cutoff energies are larger for smaller work functions. In general, for high PE yield, the second term in Eq. (3) dominates, leading to larger cutoff energies. However, for the low PE yield in Figure 4(e), the second term in Eq. (3) becomes negligible and plasmonic enhancement dominates. Since the plasmonic field enhancement is slightly larger for gold than for silver, the cutoff energy is slightly higher for gold than for silver, especially at high intensities.

**Figure 5:**
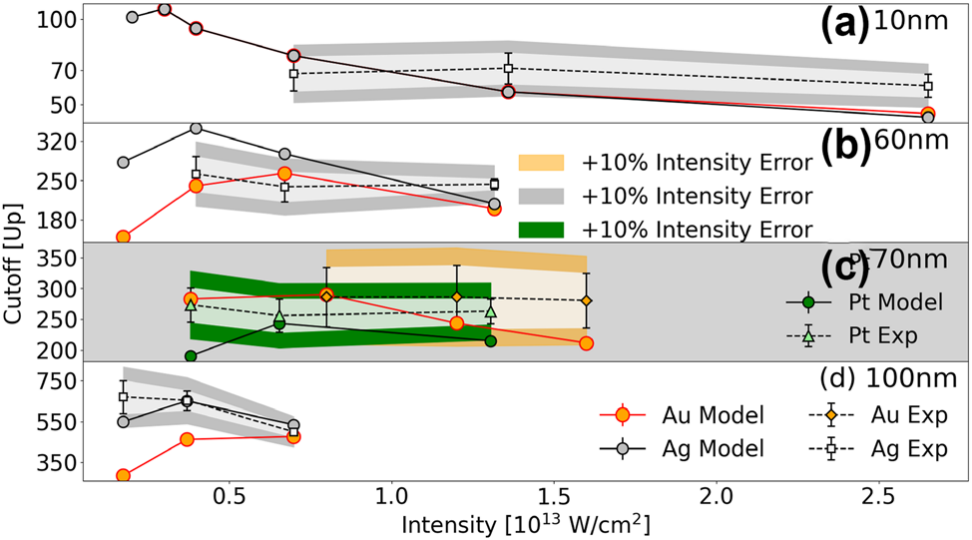
Simulated and experimental PE cutoff energies in Figures 4a–d in units of the incident-laser ponderomotive energy *U*_*p*_(*I*_0_) ∼ *I*_0_.

Figure 5 compares the simulated and experimental cutoff energies in Figure 4, scaled by the incident-laser ponderomotive energy *U*_*p*_(*I*_0_). The error bars for the scaled cutoff energy are interpolated and shown as light- and dark-colored bands. They are calculated as

(5) $$\Delta E_{Up}=|\frac{\Delta E_{eV}}{Up}|+E_{Up}|\frac{\Delta I_{0}}{I_{0}}|,$$

where Δ*E*_*eV*_ represents the uncertainty in the cutoff energy in units of eV. The light shaded regions in Figure 5 correspond to the error range given by the first term in Eq. (5), representing the uncertainty in the *U*_*p*_–scaled cutoff energy. The darker shaded regions additionally account for a 10 % uncertainty in the laser peak intensity, as described by the second term in Eq. (5). This conservatively assumed accuracy of 10 % is of the order of the estimated intensity accuracy in our experiment (cf., Section 2.2) and clearly exceeds the typical calibration standard for laser peak intensities of 1.3 % [72]. The lighter regions exclude this intensity-related uncertainty.

In striking contrast to atomic targets, cutoff energies for NPs are significantly higher and exhibit a nonlinear dependence on the laser peak intensity *I*_0_. *U*_*p*_–scaled cutoffs reach maxima that vary with the size and composition of the NPs. The emission saturation effect discussed in Section 3.1 occurs with increasing laser peak intensity *I*_0_. At low laser peak intensity and far from the above-introduced emission saturation intensity, increasing the laser peak intensity leads to a rapid increase in yield and, consequently, an increase in the cutoff energies in Figures 4 and 5. In contrast, at high laser peak intensity and close to the emission saturation intensity, a further increase in the intensity results in a slow yield increase and, consequently, an increase in the cutoff energy in units of eV in Figure 4 and decreasing *U*_*p*_-scaled cutoffs in Figure 5.

For the considered transition metals and NPs of the same size, the simulated cutoff energies tend to converge to the same value at laser high intensity. The reason for this behavior is that – at high intensities – differences in the relevant material properties, i.e., different dielectric responses (resulting in material dependent plasmonic field enhancements) and different work functions (accounting for different net PE yields), become negligible, since these intensities are very close to the emission saturation intensity. In other words, at such high intensities, residual-charge and repulsive-Coulomb interactions between a large number of PEs become the primary elementary interactions that determine the shape of the PE spectrum at high PE energies and thus the cutoff energy.

A similar convergence to intensity-independent cutoff energies was observed for dielectric NPs [22]. The authors’ numerical simulations show a decreasing intensity and material dependence of the *U*_*p*_–scaled cutoff energy due to dominant many-particle interactions, similar to our finding, albeit at larger laser intensities. The onset of this saturation effect occurring at larger intensities for dielectric, as compared to metal, NPs is expected, since higher field strengths are required for dielectric materials to release sufficiently many electrons for generating overwhelming PE Coulomb interactions.

In view of the PE spectra and cutoff energies discussed in this work being determined by complex strong-field driven many-electron interactions [26], the main characteristics in our measured spectra and cutoff energies and trends in their dependence on the NP size and laser-intensity are reasonably well reproduced by our numerical simulation, while some discrepancies remain. An important uncertainty in our simulation derives from our use of modified Fowler-Nordheim tunneling rates [26], as mentioned above. A more realistic account of electron tunneling at the NP surface, possibly based on density-functional theory, will likely be a key improvement for simulating more accurate PE yields. Expanding on the discussion of experimental errors given above in relation to the cutoff energies in Figure 4, we note that detector saturation decreases the reliability of the low-energy portion of our spectra. While this portion of the simulation data was truncated to allow for a better comparison with the experiment, the detection uncertainty due to saturation is not completely removed and tends to affect predominantly measurements with the largest NPs and highest laser peak intensity presented in this work, due to larger numbers of emitted PE per laser shot [49]. Furthermore, the unequal PE yield for different NP sizes imposed a practical limit on the laser intensities. Small 10 nm NPs produce a much smaller PE yield than 100 nm NPs. We therefore increased the laser intensity for the small particles, as the signal for the lowest intensity was insufficient to generate reliable VMI spectra for the determination of cutoff energies. In contrast, for the larger particles, our larger intensities saturate the MCP, rendering the recorded VMI spectra useless for determining cutoff energies. These experimental constraints lead to the seemingly large range of, in part unequal, intensities we selected for different nanoparticle sizes.

## 4 Conclusions

We measured and numerically simulated angle- and energy-resolved PE spectra for strong-field ionization from prototypical plasmonic metal nanospheres. Our experimental and simulated results reveal a complex interplay of PE emission, propagation, recombination, and rescattering. Enhanced by strong plasmonic fields, a substantial number of PEs tunnel-ionize from metal NPs, resulting in high PE yields and cutoff energies. We analyzed the dependence of PE angular distributions, yields, and cutoff energies on material type, NP size, and laser intensity for direct and rescattered photoemission. Our findings for three transition metals indicate that metal NPs are effective sources for generating high-energy PEs, with their yield and energy being tunable through target properties such as size and material type, as well as by adjusting the laser wavelength and peak intensity. In analogy to the behavior of PE cutoff energies for strong-field emission from dielectric NPs [22], at higher intensities our measured and simulated PE cutoff energies tend to converge to a metal-independent limit. Our results show that the work function, plasmonic field enhancement, and size are crucial determinants for strong-field photoemission from NPs. At high laser peak intensities, our numerical model further predicts the PE yield and cutoff energy to saturate due to the accumulation of residual charge on the NP surface.

## Supplementary Material

Supplementary Material Details
