# Supplementary material for: Generation of fast photoelectrons in strong-field emission from metal nanoparticles
Source: Nanophotonics. 2025 Apr 4;14(9):1355–64. doi: 10.1515/nanoph-2024-0719 (PMC12038575; doi:10.1515/nanoph-2024-0719)
Supplement: Supplementary file 1 — Supplementary Material Details [file j_nanoph-2024-0719_suppl_001.pdf]

Supplementary Material

S1 Intensity-dependent VMI spectra for 100 nm silver nanospheres

Figure S1 shows simulated and experimental VMI spectra for 100 nm diameter silver nanospheres.

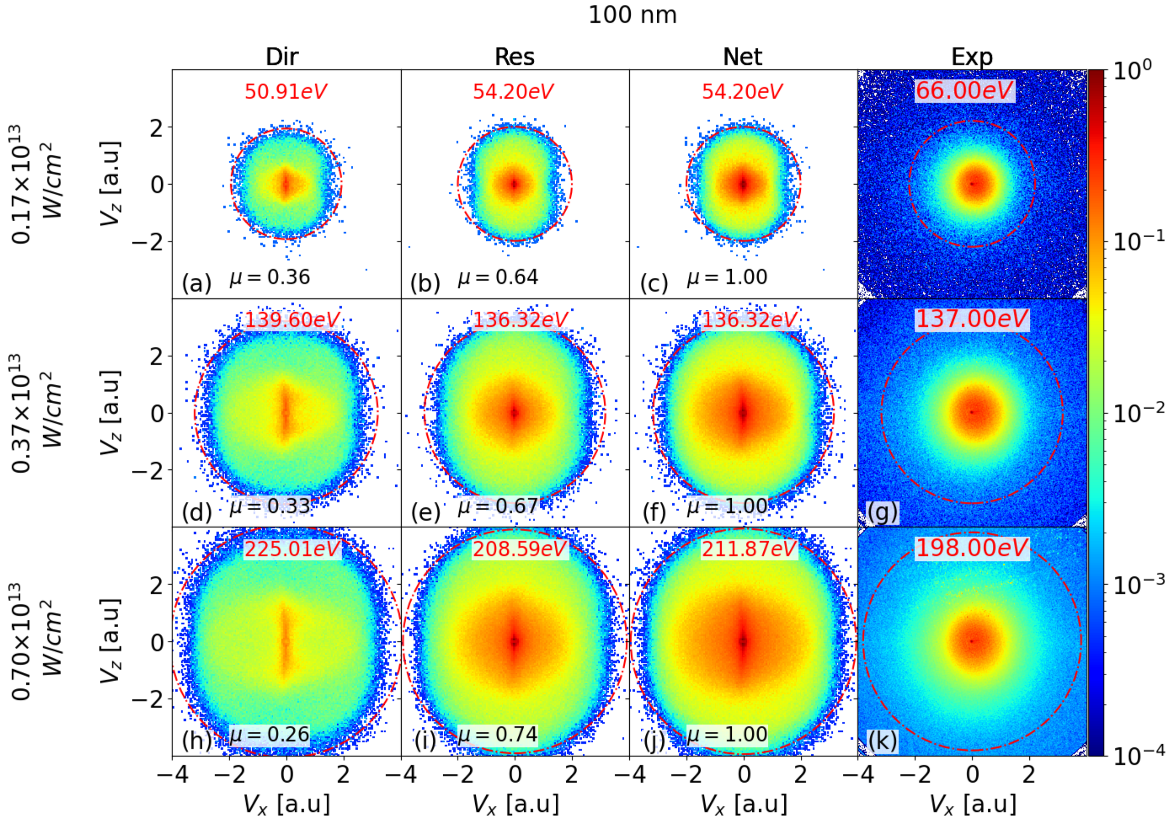

Fig. S1: As Fig. 2 in the main text for 100 nm diameter silver nanospheres and laser peak intensities of 1.7×10^{12}, 3.7×10^{12}, and 7.0×10^{12} W/cm^2 (first - third row, respectively). The laser-pulse length and wavelength are 25 fs and 780 nm. In each row, μ is the integrated PE yield, normalized to the integrated net yield in the third column. The red circle on each VMI map represents the cutoff. The value of the cutoff energy is displayed in red above each VMI graph.

S2 Intensity- and size-dependent VMI spectra for gold nanospheres

Figures S2, S3, and S4 show simulated VMI spectra for Au nanospheres with diameters of 10, 60, and 100 nm respectively.

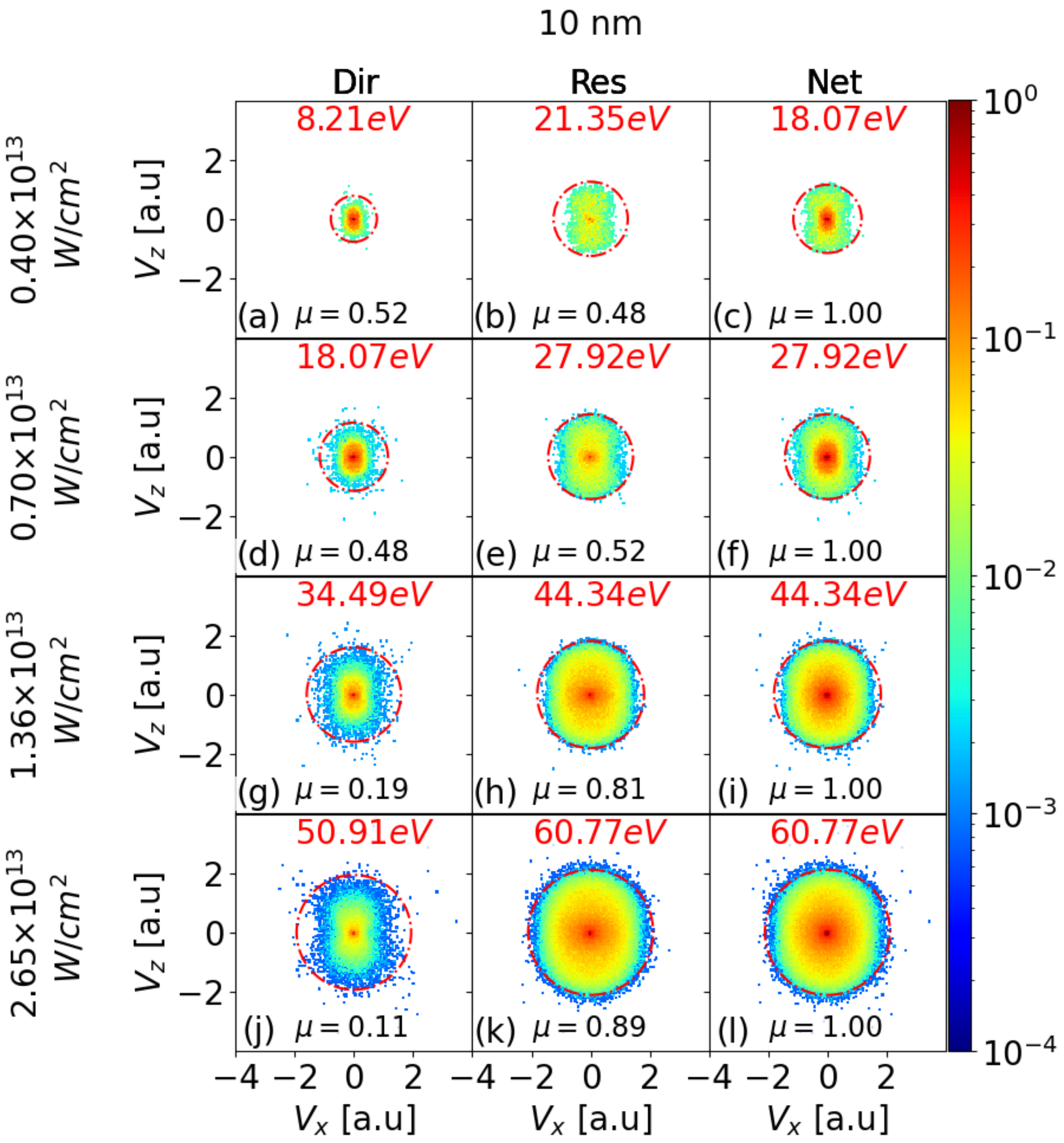

Fig. S2: As the numerical results in Fig. 2 in the main text for gold nanospheres.

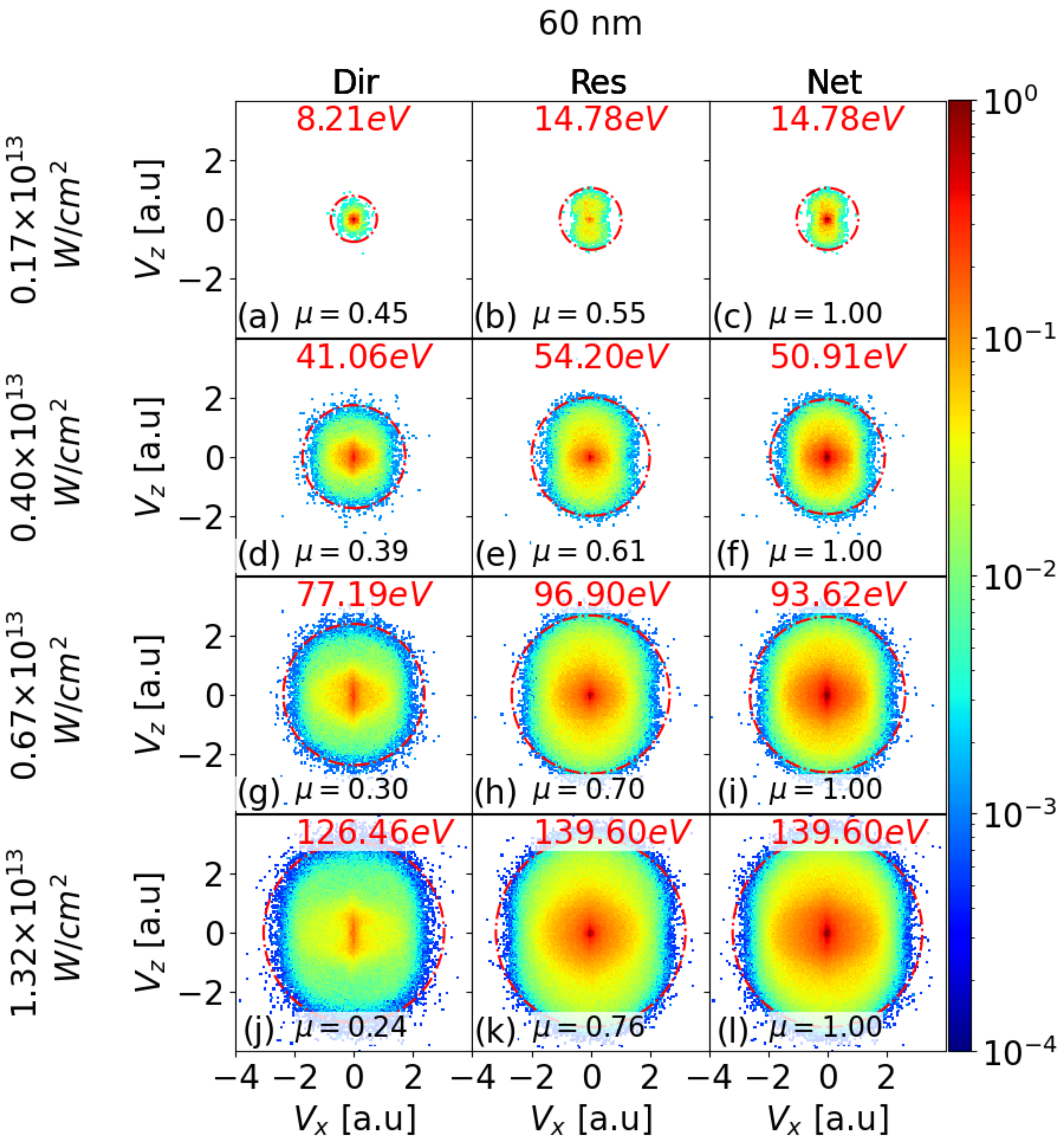

Fig. S3: As the numerical results in Fig. 3 in the main text for gold nanospheres

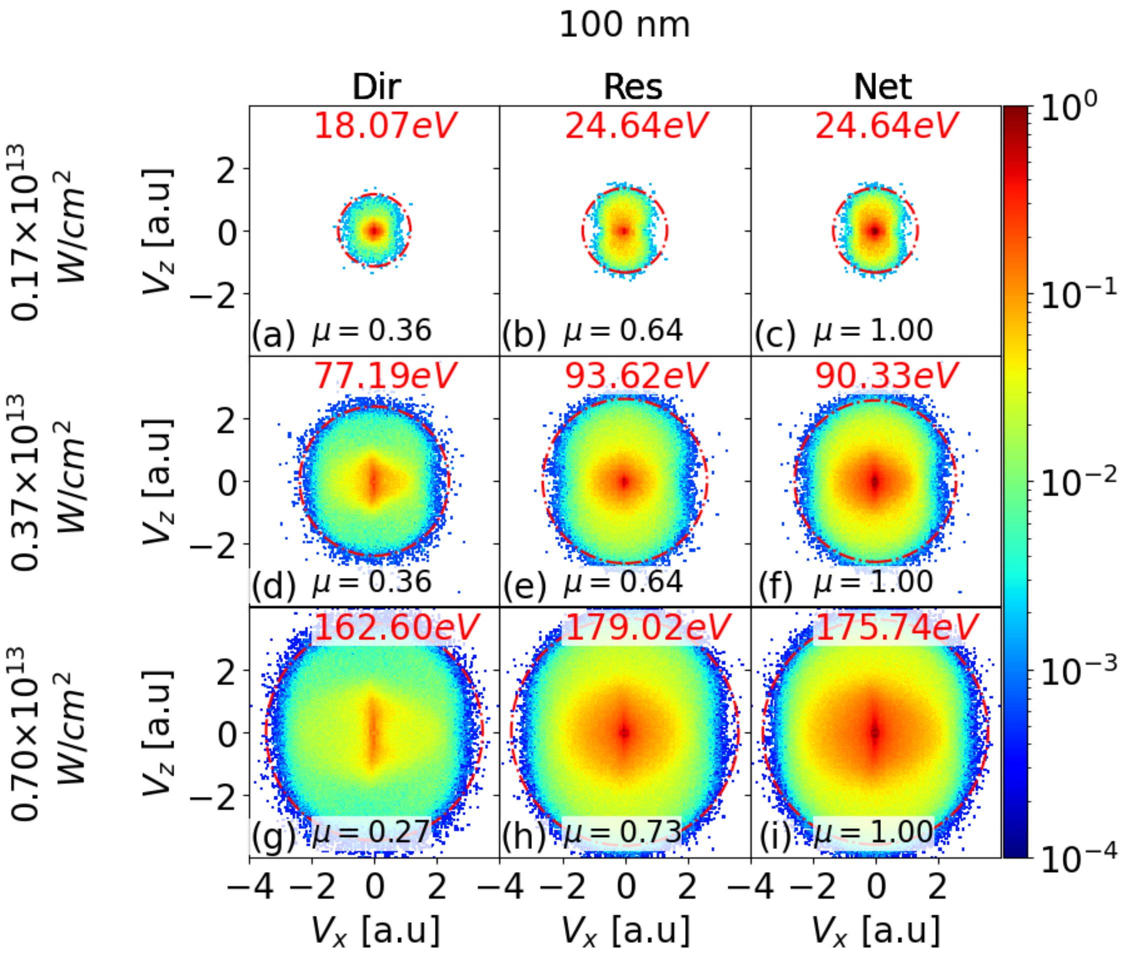

Fig. S4: As the numerical results in Fig. S1 in the main text for gold nanospheres

Figure S5 shows simulated and experimental VMI spectra for gold NPs with a diameter of 70 nm.

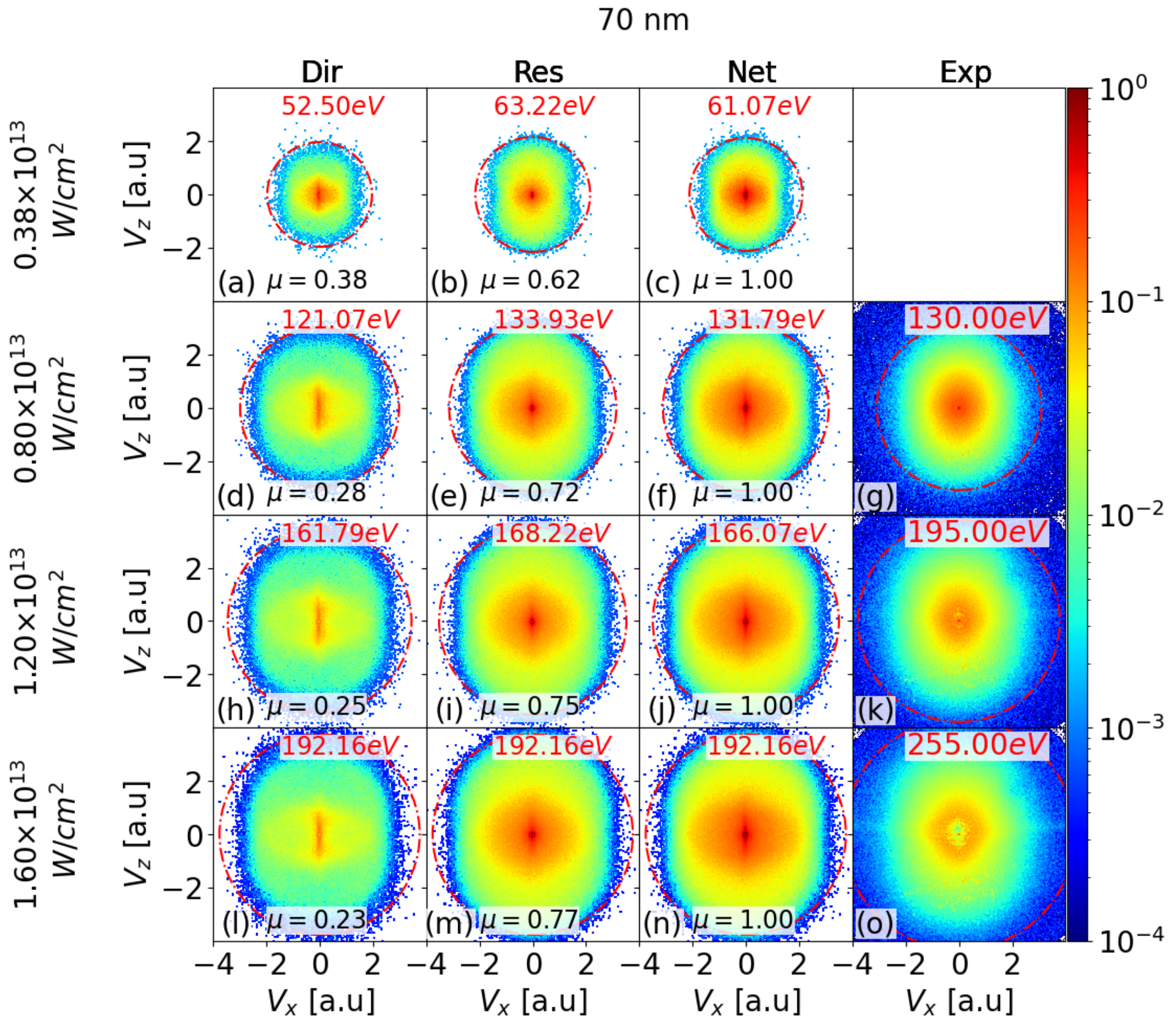

Fig. S5: Comparison of simulated direct (first column), rescattered (second column) and net (i.e., including direct and rescattered yields, third column) PE VMI spectra for gold nanospheres with a diameter of 70 nm and laser peak intensities of $I_0 = 3.8 \times 10^{12}$, 8.0×10^{12}, 1.2×10^{13}, and 1.6×10^{13} W/cm^2 (first - forth row, respectively).

S3 Intensity- and size-dependent VMI spectra for platinum nanospheres

Figure S6 shows simulated and experimental VMI spectra for platinum nanospheres with a diameter of 70 nm.

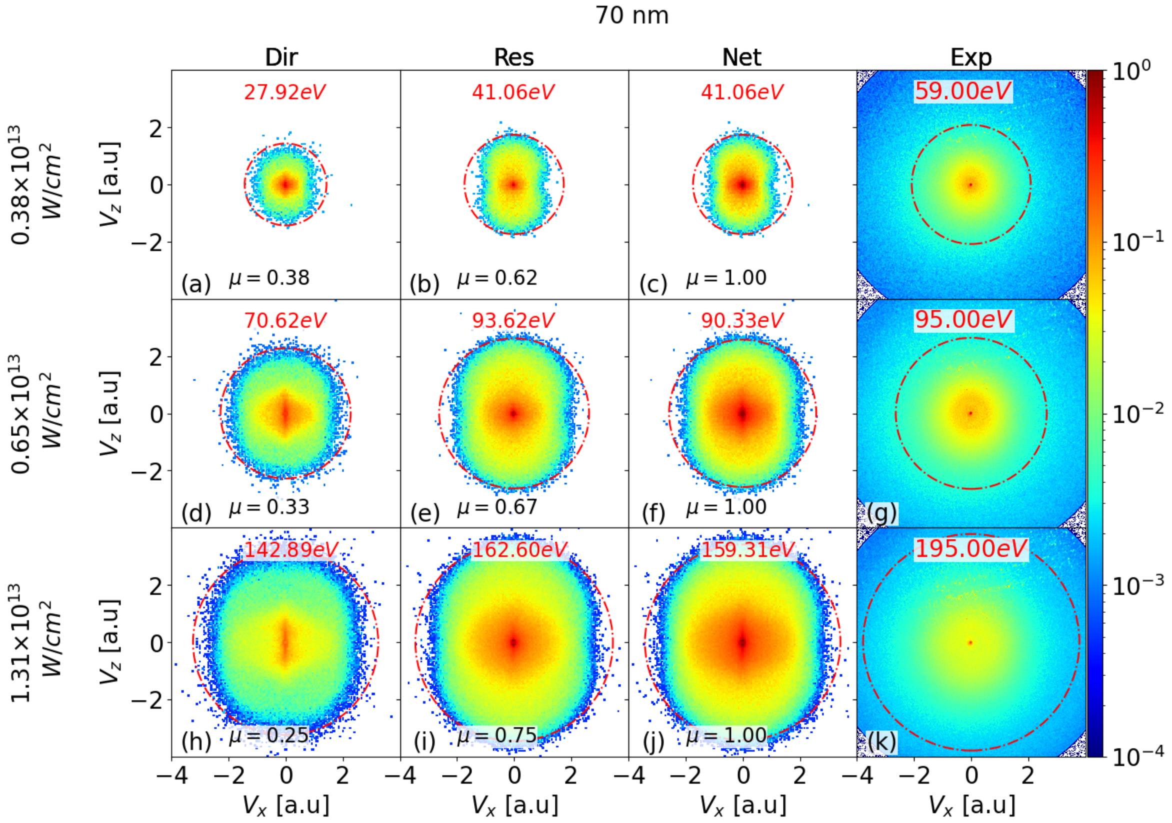

Fig. S6: Comparison of simulated direct (first column), rescattered (second column), and net (i.e., including direct and rescattered yields, third column) PE VMI spectra for platinum nanospheres with a diameter of 70 nm and laser peak intensities of $I_0 = 3.8 \times 10^{12}$, 6.5×10^{12}, and 1.31×10^{13} W/cm^2 (first - third row, respectively).
